# An Update on Ocular Surface Epithelial Stem Cells: Cornea and Conjunctiva

**DOI:** 10.1155/2015/601731

**Published:** 2015-06-04

**Authors:** Tiago Ramos, Deborah Scott, Sajjad Ahmad

**Affiliations:** ^1^Department of Eye and Vision Science, Institute of Ageing and Chronic Disease, University of Liverpool, Liverpool L69 3GA, UK; ^2^St. Paul's Eye Unit, Royal Liverpool University Hospital, Liverpool, UK

## Abstract

The human ocular surface (front surface of the eye) is formed by two different types of epithelia: the corneal epithelium centrally and the conjunctival epithelium that surrounds this. These two epithelia are maintained by different stem cell populations (limbal stem cells for the corneal epithelium and the conjunctival epithelial stem cells). In this review, we provide an update on our understanding of these epithelia and their stem cells systems, including embryology, new markers, and controversy around the location of these stem cells. We also provide an update on the translation of this understanding into clinical applications for the treatment of debilitating ocular surface diseases.

## 1. Introduction

**Figure 1:**
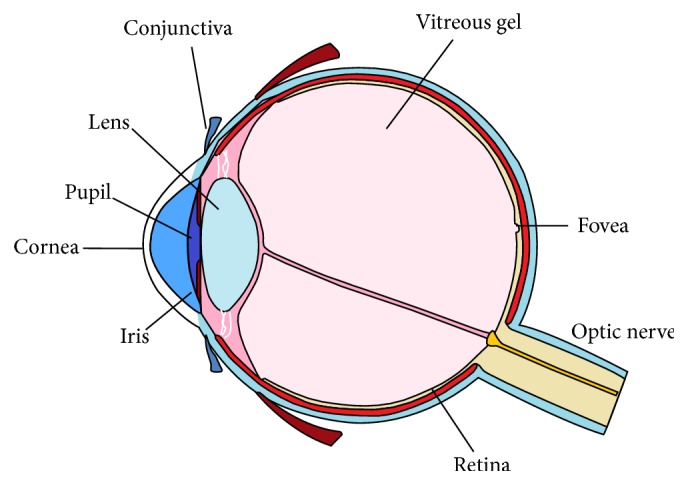
Cross-sectional illustrative view of the adult human eye.

The front of the human eye is formed by the clear cornea centrally and the white sclera peripherally (Figure 1). The cornea and sclera are covered by the corneal and conjunctival epithelia, respectively. These epithelia are nonkeratinized and stratified structures. The corneal epithelium is 5–7 cell layers thick whereas the conjunctival epithelium is 3–5 cell layers thick. These two epithelial structures are bathed in a tear film, and together these form the front surface of the eye (the ocular surface) [1, 2]. An important function of the ocular surface (cornea, conjunctiva, and the overlying tear film) is to protect the eye from injury, infection, and desiccation [2]. The cornea is a clear structure that is composed of five main layers and its main function is to transmit and focus light into the eye. The superficial layers of the cornea (the stratified epithelium, Bowman's layer, and the superficial stroma) are continuous with the conjunctiva. The conjunctiva is a thin loose transparent mucous layer covering the anterior surface of the globe and the posterior surface of the eyelids [2]. The conjunctiva is divided into three regions: bulbar (covering the surface of the eye), palpebral (lining the undersurface of the eyelids), and the forniceal region in between. The conjunctival epithelium contains superficial scattered goblet cells that produce important mucins for the tear film. Physically separating the corneal epithelium and the conjunctival epithelium is a narrow band of limbal epithelium that encircles the cornea. The limbal epithelium acts as a barrier between the clear avascular cornea centrally and the opaque vascularized conjunctiva peripherally. The limbal epithelium also contains the stem cells (SCs) that renew the corneal epithelium, the limbal SCs (LSCs) [3, 4].

Both corneal and conjunctival epithelia are susceptible to a wide range of diseases from injuries such as chemical burns to inflammatory diseases such as mucous membrane pemphigoid and Stevens-Johnson syndrome. These can result in significant visual impairment and ocular surface pain. Different treatment modalities have already been presented as a therapy for limbal SC deficiency (LSCD) with significant clinical improvements [5, 6]. However, the approaches for developing conjunctival epithelial constructs for scarring conjunctival diseases are still very limited [7].

In the present review we provide a concise update on ocular surface epithelial SCs: their location, distribution, and the markers used to identify them. The clinical applications of corneal and conjunctival epithelial and their SCs will also be discussed.

## 2. Embryological Origins of the Ocular Surface Epithelia

In order for us to understand ocular surface epithelial SC biology, it is important to understand the embryological origin of the corneal epithelium and the conjunctival epithelium. It has been shown that these two cell lineages arise simultaneously from Pax6^+^ ectodermal cells that remain on the embryonic ectodermal surface of the developing eye once the lens vesicle has formed [8, 9]. The* PAX6* gene encodes a transcription factor critical for normal embryonic development. The PAX6 protein is expressed in the developing eye, multiple brain regions, olfactory bulb, neural tube, gut, and pancreas [10]. In humans, insufficient PAX6 protein expression results in severe congenital defects of the eye [11]. It is therefore considered the master gene for oculogenesis.

The developing human corneal epithelium is first apparent at 6 weeks after ovulation [12]. The primitive corneal epithelium is initially composed of two cell layers (as compared to the five to seven layers in the adult). This primitive epithelium is also responsible for forming a prominent primary acellular corneal stroma and Bowman's layer [13]. Sometime between 8 weeks of gestation (when the eyelids fuse together) and 26 weeks of gestation (when eyelids open), the corneal epithelium stratifies to four to five cell layers thick. Adhesion complexes only become detectable by 19 weeks of gestation. The further development in utero leads to an increase in the number of hemidesmosomes, an increase in the fibril penetration into Bowman's layer, and an increase in Bowman's layer thickness. Maturation of the corneal epithelium is therefore related to eyelid development [12].

Despite their closeness, the corneal ECs and the conjunctival ECs belong to distinct lineages [14, 15] arising from different cell populations [16].* In vivo *studies in rabbit have shown that limbal and corneal EC derived cysts contained only stratified squamous-type ECs. In contrast, conjunctival EC derived cysts contained stratified columnar-type ECs interspersed with periodic acid-Schiff (PAS) staining cells (PAS is a mucin stain) with a goblet-like structure (the goblet cells) [16]. This supports the hypothesis that corneal and limbal ECs originate along a different embryonic lineage to conjunctival ECs, and that the goblet cells originate from the conjunctival compartment.

## 3. Conjunctival Epithelium and Its Stem Cells

### 3.1. Location of Conjunctival Stem Cells (CjSCs)

Whilst it is commonly regarded that the SCs for the corneal epithelium (the LSCs) are located in the limbal epithelium, the location of SCs for the conjunctival epithelium is more controversial. The following regions of the conjunctiva have been proposed as possible sites for the SCs: the fornix region [17], the limbus [18], bulbar conjunctiva [19], palpebral conjunctiva [20], and at the mucocutaneous junction on the eyelid margin [21]. Bromodeoxyuridine label retention (a property of quiescent SCs) in GFP-labelled mice suggests that epithelial SCs in the conjunctiva are uniformly distributed throughout the whole surface [22]. Another important property of epithelial SCs is their ability to initiate clonal growth* in vitro* and yield colonies consisting of small cells that have a long survival time (called holoclones). By analyzing epithelial colonies cultured from different regions of the human conjunctiva, Pellegrini et al. were the first to suggest that conjunctival epithelial SCs are located uniformly in the bulbar conjunctiva and the fornices [19]. More recently, Stewart et al. using both clonogenic ability and expression of putative SCs markers (ABCG2 and p63) showed that the medial canthal and the inferior forniceal areas are the preferred area for the human CjSCs. They suggested that those areas provide greater physical protection but more importantly are especially rich in goblet cells, intraepithelial mucous crypts, blood vessels, melanocytes, and immune cells, features shared with other SC niches [23]. In other studies, where the slow cell-cycling and the great proliferative potential were analyzed it has been shown that the forniceal region of the rabbit conjunctiva contains the largest proportions of cells with high proliferative potential and a higher percentage of slow cycling cells (14% of ECs) than the bulbar (5%) and palpebral conjunctiva (1%) [15, 17].

Although there are no specific markers for CjSCs, ABCG2 positive cells have been found in clusters of human bulbar conjunctival epithelial basal cells and these cells display many features that are consistent with the epithelial SC phenotype, slow cycling, clonogenic capacity, and resistance to phorbol-induced differentiation [24]. Other immunohistological studies also suggest the presence of p63 and ABCG2 positive cells in the bulbar conjunctival epithelium [25]. Clinical observations also indicate that the CjSCs are located in the fornix and/or in the bulbar conjunctiva [26].

Despite the different observations, resulting from differing techniques and species used, all seem to point in the higher amount of conjunctival SCs in the forniceal area. The fornix may provide greater physical protection, intraepithelial mucous crypts, vasculature, and immune cells, features shared with other SCs niches [23].

### 3.2. Markers for Conjunctival Epithelium and Its SCs

The identification of a marker that is expressed in the conjunctival epithelium but not in the corneal epithelium has been a growing need. Because of their different patterns of expression, cytokeratins (CKs) have been widely used to distinguish the different ECs of the ocular surface [27] (Table 1). CKs are intermediate filament-forming proteins responsible for the structural integrity and function of ECs [28]. Different CKs have been suggested as specific markers for conjunctival ECs, amongst those are CK4, CK13, CK19, and CK15.

*CK4*. In mouse conjunctiva, CK4 is expressed in all the epithelial layers, but the expression in the basal layers seems to be weaker and more focal. However, CK4 is also expressed in mouse and rabbit superficial layers of the corneal epithelium* in vivo* as well [29, 30].

*CK13 and CK19*. CK13 and CK19 are the most accepted markers for conjunctival ECs with confounding results arising from different investigations. Ramirez-Miranda et al. have shown that CK13 and CK19 expression is significantly increased in the human conjunctiva in comparison to the human cornea. They have shown that CK13 is expressed only in the suprabasal limbal epithelium and in all layers of the conjunctival epithelia and it is completely absent in the cornea. On the other hand, CK19 is present at substantial levels in the peripheral corneal epithelium and in all layers of the limbus and conjunctiva epithelium [28, 31, 32]. They showed that none of the CK12-positive cells expressed CK13 in the central cornea and the limbal ECs expressed either CK12 or CK13 (CK12 is known to be a specific marker for corneal EC; see Section 4.2). On other hand, CK12 and CK19 positive cells were colocated throughout the limbus and peripheral cornea suggesting a higher specificity of CK13 for conjunctival ECs [28].

*CK15*. Other investigators have also studied the expression pattern of CK15, a minor cytoskeletal component of stratified tissue proposed to be a marker for progenitor cells [33]. All studies revealed that CK15 is expressed in the basal layers of the limbal and conjunctival epithelia but it is absent in the corneal epithelium [34, 35]. Other* in vitro *studies have also shown the expression of CK15 by conjunctival epithelial progenitor cells but they suggest that more differentiated cells may also express this marker [36].

Other markers for conjunctival epithelial cells are the mucins. Epithelial MUCs are a heterogeneous group of large glycosylated proteins, which form the viscous, gel-like mucous layer of the tear film. Both MUC1 and MUC5AC have been postulated as being markers for conjunctival ECs and/or goblet cells.

*MUC1*. The exclusivity of MUC1 to conjunctival ECs is debated with some proposing it as a conjunctival epithelial specific marker [37] and others suggesting expression throughout the entire ocular surface in health [38, 39].

*MUC5AC*. MUC5AC, on the other hand, has long been postulated as being specific to the conjunctival goblet cells [40]. Although it is not detected in the ECs of the conjunctiva, it is the best surrogate marker for the presence of conjunctival epithelium by identifying the goblet cells that are absent in the corneal epithelium in health. Another method for identifying goblet cells is by PAS staining of MUCs. This is indeed used clinically in the investigation of corneal conjunctivalisation by corneal impression cytology [40].

There are few studies looking at identifying markers for CjSCs. The most commons are ATP-binding cassette subfamily G member 2 (ABCG2) and the transcription factor p63. ABCG2 is a cell surface transmembrane transporter that is present in many adult SCs, including LSCs. Conceptually, it may form a component of the molecular mechanisms by which long-lived SCs reduce the potential for genomic damage over their extended lives, and their expression has been correlated with SC activity [41]. In human conjunctiva its expression has been found in clusters of basal [24], medial canthal and the inferior forniceal areas conjunctival ECs [23]. Those ABCG2 positive cells display many features that are consistent with the epithelial SC phenotype, slow cycling, clonogenic capacity, and resistance to phorbol-induced differentiation. p63 is a transcription factor that is known to be expressed by LSCs and early transient amplified cells [42–44]. Stewart et al. using cytochemistry analysis showed a preferred location of this transcription factor in the inferior forniceal and medial canthal. The cells in those areas exhibited the higher clonogenic capacities which suggest a phenotype that is consistent with SCs [23].

## 4. Limbal SCs

### 4.1. Location of Limbal SCs

**Figure 2:**
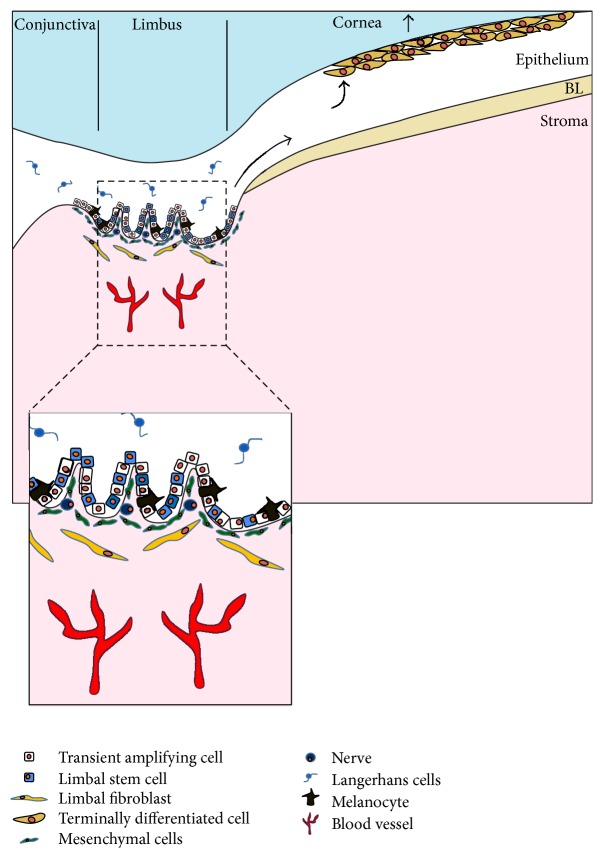
Limbal SCs for the corneal epithelium reside in the basal layer of the limbal epithelium. Transient amplifying cells divide and migrate towards the central cornea to replace the terminal corneal ECs that get shed from the corneal surface. The stroma of the limbal SC niche is populated by fibroblasts and nourished by blood vessels (BL: Bowman's layer).

In corneal epithelial homeostasis the epithelium is constantly renewed to replace desquamating cells that are shed from the corneal surface (Figure 2). This homeostasis is outlined in the XYZ hypothesis of corneal epithelial maintenance [45]. In brief, this describes the proliferation and motility of basal ECs at the periphery of the cornea centripetally along the basement membrane towards the center of the cornea (component X) and then the movement of cells from the basal layers to the superficial corneal epithelial layers (component Y) to replace the ECs that are shed from the corneal surface (component Z).

It is widely accepted that the corneal epithelial equilibrium described above is maintained by the self-renewing undifferentiated SCs located at the limbus. A consistent body of evidence demonstrates the limbal location of corneal epithelial SCs with the original studies dating back to the late 1980s [46]. In brief, the evidence for this includes the following: the presence of basal limbal ECs with a high nucleus to cytoplasm ratio and putative protein expression consistent with SCs [47, 48], using DNA-labelling and cell-cycling studies, the presence of limbal ECs in a quiescent state but with a high proliferative response in injury; and clinical observations on limbal and corneal epithelial wound healing in humans.

Recent studies in animal models however suggest that there is also a reservoir of SCs within the corneal epithelium itself in addition to the LSCs. Recent nonhuman studies have shown corneal epithelial maintenance without limbal input and survival and self-maintenance of SCs outside the limbal SC niche [49]. Successful corneal epithelial regeneration by sequential corneal epithelial transplantation in a murine model was first proposed as evidence for this [50]. Recently, again in a murine model, lineage-tracing experiments have shown that although the limbus is the prime source for corneal epithelial maintenance, there is also a reservoir of clonogenic cells within the corneal epithelium itself [51]. In addition to these studies, 48-week follow-up rabbit studies have shown that although normal corneal epithelium cannot initially be maintained following the removal of limbal epithelium, there is evidence of corneal epithelial normalization at 48 weeks [52]. Bringing together all the evidence available, although corneal epithelial maintenance relies upon SCs within the limbus, there is some evidence in animal models that there may also be a pool of progenitor cells within the corneal epithelium itself when homeostasis is compromised [53]. Further research is required particularly in human studies to provide strength and consistency to this theory.

### 4.2. Markers for Limbal SCs

Despite decades of research, no definitive marker for LSCs has been identified. Comprehensive reviews of putative suggested LSC markers have been published elsewhere [54, 55]. In brief, putative positive markers include the transcription factor ΔNp63*α* and the ABCG2; whilst negative markers include CK3 and CK12, the structural proteins found in the corneal epithelium. More recently, murine and human studies have shown that cells expressing the ATP-binding cassette, subfamily B, member 5 (ABCB5) are required for corneal epithelial homeostasis and repair [56]. They showed that ABCB5 expressing cells were localized to the limbus and coexpressed ΔNp63*α* but not CK12.

## 5. The Clinical Applications of Corneal and Conjunctival Epithelia and Their SCs

Diseases of the corneal and conjunctival epithelia result in debilitating and blinding eye diseases that are often chronically painful. These diseases result from injury such as burns, inflammatory and immune mediated diseases (such as mucous membrane pemphigoid), and iatrogenic causes (such as radiotherapy and chemotherapy). Our understanding of corneal and conjunctival epithelial biology and increasing knowledge of their SCs is critical to the clinical management of these diseases.

Limbal SC failure results in an abnormally regenerating corneal epithelium that then gets replaced by a more opaque conjunctival epithelium and blood vessels. This results in loss of vision. Transplanting healthy limbal tissue or cultured limbal epithelium containing limbal SCs has been shown in numerous studies to result in normalization of the corneal epithelium [5, 6]. Using DNA fingerprinting methods in allogeneic transplants, it has been shown that often the donor cells do not survive on the corneal surface despite the corneal epithelium regenerating normally [57]. This then begs the question as to how the transplanted limbal epithelium and limbal SCs contribute to normalization of the host corneal epithelium. Many now believe that paracrine influences from the transplanted ECs may result in host SC recovery or certainly contribute to it. It is also interesting to note that the success rate for cultured limbal epithelial transplants is approximately equal for both allogeneic and autologous transplants (about 75% success) [58]. This may suggest that transplant failure is less likely to result from rejection and that other factors may well be at play (such as the number of limbal SCs in the transplanted tissue).

As outlined above, although improved knowledge of limbal SC biology has resulted in significant advances in clinical benefit, it has been slower with regard to the conjunctival epithelium. However, our understanding of conjunctival epithelial SCs and their culture has improved. Many groups are now working on developing conjunctival epithelial constructs for scarring conjunctival diseases with still very limited results [7]. Regarding conjunctival epithelium, as our understanding of conjunctival epithelial SCs improves, clinical applications of this biology will bring considerable benefit. There are already several groups working on developing conjunctival epithelial constructs for scarring conjunctival diseases. It has even been suggested that cultivated conjunctival epithelium may be used as a treatment for limbal SC and corneal epithelial failure [7].

## 6. Conclusion

Our knowledge of limbal SC and conjunctival epithelial SC biology has progressed considerably in the last two decades with significant clinical advances being made in limbal SC transplantation. There however remain critical areas for further work. These include more understanding of the cellular biology of these SCs and in the process the identification of further markers. This will enable the development of nonsurgical approaches (drug and biological agents) to the management of ocular surface epithelial diseases. In addition, the location of these SCs is important to understand fully. There are emerging concepts that are beginning to challenge our conventional understanding of this.

## Figures and Tables

**Table 1:** A table showing the distribution of different keratins across the ocular surface epithelia (corneal, limbal, and conjunctival).

Cytokeratin	Cornea	Limbus	Conjunctiva
CK1, 2, 10	+	+	++

CK3	Periphery: basal and intermediate cells +++ Centre: all cells ++	+++	++

CK5	+++	+++	Basal cells +++, intermediate cells ++

CK14–18	+++	Basal and superficial cells +++	+++

CK1–8 and K5, 10, 11	+++	+++	+++

CK8, 18, 19	+++	Superficial and intermediate cells ++	+++

## Abbreviations

SC: Stem cell
EC: Epithelial cell
LSC: Limbal stem cells
CjSC: Conjunctival stem cell
CK: Cytokeratin
MUC: Mucin.
